# Insight into the labeling mechanism of acceleration selective arterial spin labeling

**DOI:** 10.1007/s10334-016-0596-6

**Published:** 2016-10-27

**Authors:** Sophie Schmid, Esben T. Petersen, Matthias J. P. Van Osch

**Affiliations:** 1 Department of Radiology, C.J. Gorter Center for High Field MRI, Leiden University Medical Center, C3-Q, Albinusdreef 2, 2333 ZA Leiden, The Netherlands; 2 Danish Research Centre for Magnetic Resonance, Copenhagen University Hospital Hvidovre, Hvidovre, Denmark; 3 Leiden Institute for Brain and Cognition, Leiden, The Netherlands

**Keywords:** Perfusion magnetic resonance imaging, Cerebrovascular circulation, Cerebrovascular disorders, Microvessels, Capillaries

## Abstract

**Objectives:**

Acceleration selective arterial spin labeling (AccASL) is a spatially non-selective labeling technique, used in traditional ASL methods, which labels spins based on their flow acceleration rather than spatial localization. The exact origin of the AccASL signal within the vasculature is not completely understood. To obtain more insight into this, the acceleration selective module was performed followed by a velocity selective module, which is used in velocity selective arterial spin labeling (VS-ASL).

**Materials and methods:**

Nine healthy volunteers were scanned with various combinations of the control and label conditions in both the acceleration and velocity selective module. The cut-off acceleration (0.59 m/s^2^) or velocity (2 cm/s) was kept constant in one module, while it was varied over a large range in the other module. With the right subtractions this resulted in AccASL, VS-ASL, combined AccASL and VS-ASL signal, and signal from one module with crushing from the other.

**Results:**

The label created with AccASL has an overlap of approximately 50% in the vascular region with VS-ASL, but also originates from smaller vessels closer to the capillaries.

**Conclusion:**

AccASL is able to label spins both in the macro- and meso-vasculature, as well as in the microvasculature.

**Electronic supplementary material:**

The online version of this article (doi:10.1007/s10334-016-0596-6) contains supplementary material, which is available to authorized users.

## Introduction

Arterial spin labeling (ASL) can be used as a noninvasive MR technique to quantify local tissue perfusion. In conventional ASL, the labeling is based on a spatial localized tagging of blood spins by either inversion or saturation in a plane proximal to the imaging region. The image is acquired after a post labeling delay (PLD), chosen approximately equal to the longitudinal relaxation (*T*_1_) of blood for cerebral perfusion imaging. This choice in PLD represents a compromise between the transport time, which should be long enough for the labeled blood to reach the tissue, and the loss of label due to *T*_1_-relaxation. However, even in healthy, young volunteers the variation in transit time within a single slice can be up to hundreds of milliseconds [1, 2]. Moreover, in elderly subjects and patients with pathological brain conditions, slow and/or collateral flow is a frequently encountered confounder of conventional ASL techniques. This can lead to severe underestimation of cerebral blood flow (CBF), because not all labels will have reached the microvascular bed during readout [2].

Recently, a new family of ASL techniques has been introduced. These spatially non-selective ASL (SNS-ASL) methods label spins by saturation based on their flow velocity (i.e. velocity-selective ASL, frequently abbreviated as VS-ASL) or acceleration (AccASL) rather than spatial localization [3, 4]. The label is generated globally, so also applies within the imaging region where perfusion is measured. Since the labeling is also much closer to the capillaries, this makes the spatially non-selective techniques more robust with respect to transit time effects. Therefore, VS-ASL can provide quantitative CBF maps even under slow and collateral flow conditions [5, 6]. Because the temporal SNR of AccASL has been found to be higher than the temporal SNR of VS-ASL [4], this new technique could be an interesting alternative SNS-ASL approach, although the exact origin of the signal is not completely understood.

In VS-ASL the velocity-selective labeling module tags all spins that flow faster than a predefined cut-off velocity, irrespective of whether these are located in arterial or venous blood. The acceleration-dependent preparation differs from a velocity-selective approach in that it does not affect the magnetization from spins flowing at a constant velocity, but saturates spins that are accelerating (or decelerating) above a certain cut-off acceleration (or deceleration) [7]. These different approaches to labeling result also in a difference in our understanding of the exact location in the vascular tree where the label is created. For VS-ASL this location can be estimated by comparing the cut-off velocity to the typical velocity within the vascular tree [8], although these velocities are expected to vary from person to person depending on, for example, age and vessel stiffness. In the vascular tree, the average velocity of the spins decreases from the arteries toward the capillaries, after which their average velocity increases slightly while flowing into the venous system. By the use of a second velocity module approximately 1.5–2.0 s after the first, an arterial label can be distinguished from a venous label and quantification of CBF becomes feasible due to the fact that the temporal width of the bolus of labeled spins is now controlled. In AccASL the label is predominantly created within the arterial side of the vasculature, where the pulsatility is known to be higher than in the veins, but at what level of the vasculature the exact origin of the signal originates is poorly understood. It has been suggested that the signal is of mixed haemodynamic origin, including both CBF and CBV-weighting, and that the label could originate from cardiac cycle fluctuations, general flow acceleration/deceleration in the vasculature, and from the tortuosity of the vessels, at both macro- and microvascular levels [9].

The aim of this study was to obtain more insight into the origin of the labelling mechanism in AccASL by combining this method with a VS-module. This will show whether both approaches label at similar locations in the vascular tree or whether AccASL labels even closer to the microvasculature than VS-ASL.

## Materials and methods

### Spatially non-selective arterial spin labeling methods

**Fig. 1:**
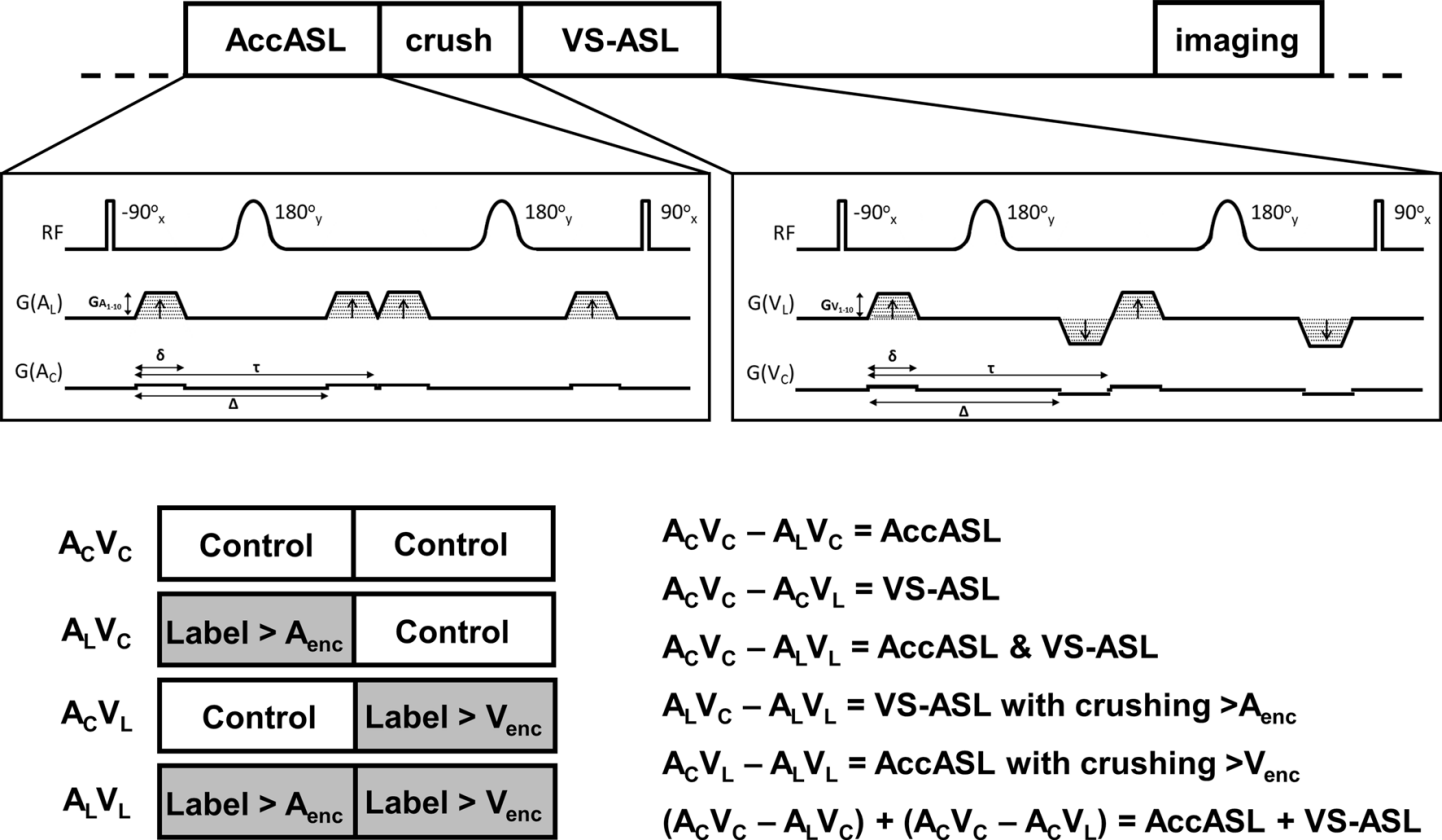
**a** A schematic representation of the sequence. The gradients in the acceleration (AccASL) and velocity (VS-ASL) selective labeling modules are described as: *G* the amplitude of the gradients, *δ* the gradient duration, *Δ* the time between the leading edges of the first two gradient lobes and *τ* the time leading edges of the first and the third gradients. **b** Four different combinations of the control and labeling conditions are possible with the acceleration- and velocity-selective labeling modules: *A*_C_ *V*_C_, *A*_L_ *V*_L_, *A*_C_ *V*_L_ and *A*_L_ *V*_C_, where *A*_C_ is the control condition of the acceleration-selective module, *V*_C_ is the control condition of the velocity-selective module, *A*_L_ is the label condition of the acceleration-selective module and *V*_L_ is the label condition of the velocity selective module. **c** By combining and subtracting the acquired scans different types of images can be calculated

The labeling modules of both AccASL and VS-ASL combine a pair of spatially nonselective hard 90° pulses with two identical adiabatic 180° refocusing pulses to correct for phase shifts due to inhomogeneities of the magnetic field and chemical shifts [10]. Motion sensitizing gradients, placed in-between these radiofrequency pulses, as shown in Fig. 1, can be used to dephase the magnetization of flowing spins, and the polarity of these gradients determines whether flowing spins with constant velocity (VS-ASL) or accelerating (AccASL) spins are affected. In the velocity-sensitive labeling module the second and fourth gradients are negative, whereas in the acceleration-sensitive labeling module all gradients are positive, inducing an effective zero first-gradient moment, giving no velocity sensitisation, but acceleration sensitisation due to a second-gradient moment [4, 11]. Spins with a flow velocity or acceleration above a cut-off velocity (*V*_enc_) or cut-off acceleration (*A*_enc_), respectively, are dephased: both parameters are defined as to correspond to a phase change of pi. The effective first-gradient moment (*m*_1_) of the velocity-sensitizing sequence can be calculated to be *m*_1_ = *G*∙*Δ*∙*δ*, with *G* the amplitude of the gradients, *δ* the gradient duration, and *Δ* the time between the leading edges of the first two gradient lobes. By varying these parameters, the cut-off velocity can be set, according to *V*_enc_ = *π*/(2*γ*∙*m*_1_), where *γ* is the gyromagnetic ratio. The acceleration-sensitizing sequence has an effective first-gradient moment of zero, giving no velocity sensitization, but a second-gradient moment (*m*_2_) of *m*_2_ = 4∙*G*∙*Δ*∙*δ*∙*τ*, with *τ* the time between the leading edges of the first and the third gradients. The relation to the cut-off acceleration is *A*_enc_ = 2*π*/(*γ*∙*m*_2_) [11]. Both acceleration and deceleration, which could be considered as negative acceleration, are targeted equally well with this sequence. It should be noted that the gradients not only encode motion but also impart a diffusion sensitivity, which becomes substantial for higher moments.

The sequence used in this study consisted of an acceleration-selective labeling module immediately followed by a set of crushing gradients consecutively in *x*-, *y*- and *z*-directions to dephase any remaining transverse magnetization, all performed with an area under the gradient of 50 mT/m∙ms. Subsequently, a velocity-selective labeling module was played out. A schematic representation of the sequence is shown in Fig. 1.

The control condition of VS-ASL and AccASL consists of the same set of RF-pulses and gradients, but gradient amplitudes of 5% of the constant cut-off are employed. Since in our combined sequence the VS- and Acc-module can each be control or label, four different combinations are possible: *A*_C_ *V*_C_, *A*_L_ *V*_L_, *A*_C_ *V*_L_ and *A*_L_ *V*_C_, where *A*_C_ is the control condition of the acceleration-selective module, *V*_C_ is the control condition of the velocity-selective module, *A*_L_ is the label condition of the acceleration-selective module and *V*_L_ is the label condition of the velocity selective module. For each combination, ten different variations in labeling velocity or acceleration were acquired.

### Subtraction of ASL images

By combining and subtracting the scans acquired with different combinations of labels and control conditions, different types of images can be calculated, as summarised in Fig. 1. The difference between *A*_C_ *V*_C_ and *A*_L_ *V*_C_ will result in a signal similar to that obtained by a normal acceleration-selective scan (AccASL), since the control condition of the VS-module is assumed not to significantly influence the longitudinal magnetization and the static spins will be excluded by subtraction. Similarly, *A*_C_ *V*_C_ minus *A*_C_ *V*_L_ will yield the signal originating from the velocity-selective module (VS-ASL). *A*_*C*_ *V*_*C*_ minus *A*_*L*_ *V*_*L*_ results in a label-signal created by joint, sequential application of the acceleration- and velocity-selective modules (AccASL & VS-ASL). Whereas, the subtraction of *A*_L_ *V*_L_ from *A*_C_ *V*_L_ will provide the signal from the Acc-module followed immediately by crushing from the VS-module (AccASL with crushing >*V*_enc_) and subtraction of *A*_L_ *V*_L_ from *A*_L_ *V*_C_ will provide the signal from the VS-module preceded by crushing from the Acc-module (VS-ASL with crushing >*A*_enc_). When the crushing is performed with a certain cut-off, the difference with the labeling without crushing indicates the overlap in labeling, i.e. when *A*_L_ *V*_L_ − *A*_C_ *V*_L_ provides much less signal than *A*_L_ *V*_C_ − *A*_C_ *V*_C_, then the acceleration module labeled, in large part, the same spins as the velocity module for that *V*_enc_.

It is important to note that the labeling modules saturate spins above the cut-off acceleration or velocity. Therefore, when some spins were already saturated by the acceleration selective labeling module, the velocity selective labeling module can never result in additional saturation of these spins, i.e. no additional label is created. Therefore, when a label would originate from different vascular regions, there should be no difference between the scan with joint, consecutive AccASL and VS-ASL labeling (*A*_C_ *V*_C_ − *A*_L_ *V*_L_) or the sum of the separate AccASL and VS-ASL scans (*A*_C_ *V*_C_ − *A*_L_ *V*_C_ + *A*_C_ *V*_C_ − *A*_C_ *V*_L_). However, when both labeling modules created labels in overlapping regions, the signal from the consecutive AccASL and VS-ASL scan should be smaller than the sum of the separate AccASL and VS-ASL scans.

### MR experiments

Two types of measurements were performed. First, the amplitude of the four acceleration-sensitizing gradients were varied in ten steps (*G* = 0–30 mT/m, *A*_enc_ = ∞−0.59 m/s^2^), while the four velocity-sensitizing gradients were kept constant (*G* = 15 mT/m, *V*_enc_ = 2 cm/s). Second, the acceleration-sensitizing gradients had constant amplitude (*G* = 30 mT/m, *A*_enc_ = 0.59 m/s^2^), while the velocity-sensitizing gradients varied in ten different values (*G* = 0–20 mT/m, *V*_enc_ = ∞–1.5 cm/s). When the gradient amplitude is zero, no labeling will occur (note that in the control condition gradients with an amplitude of 5% of the constant cut-off are employed [12]). For all measurements *δ* = 1 ms, *Δ* = 17.5 ms and *τ* = 18.9 ms and the motion sensitizing gradients were encoded only in the *z*-direction. The variable gradient amplitudes are shown in Table 1.

**Table 1:** Values of the gradient amplitudes (*G*) and the corresponding cut-off velocities (*V*_enc_, top rows) and cut-off accelerations (*A*_enc_, bottom rows) for the measurement combined with respectively constant acceleration-sensitizing gradients (*δ* = 1 ms, *Δ* = 17.5 ms, *τ* = 18.9 ms, *G* = 30 mT/m, *A*_enc_ = 0.59 m/s^2^) and velocity-sensitizing gradients (*δ* = 1 ms, *Δ* = 17.5 ms, *τ* = 18.9 ms, *G* = 20 mT/m, *V*_enc_ = 1.5 cm/s)

*G* (mT/m)	0	1.25	2.5	5	7.5	10	12.5	15	17.5	20
*V*_enc_ (cm/s)	∞	24	12	6.0	4.0	3.0	2.4	2.0	1.7	1.5

*G* (mT/m)	0	2.5	5	10	15	20	22.5	25	27.5	30
*A*_enc_ (m/s^2^)	∞	7.1	3.5	1.8	1.2	0.89	0.79	0.71	0.64	0.59

A total of 9 healthy volunteers [5 males and 4 females, mean age 29 (21–63) years] participated in this study and written informed consent was obtained from all individual participants included in the study. This study was part of a project for protocol development as approved by the local Institutional research board. All scans were performed on a 3 T scanner (Philips Healthcare, Best, The Netherlands) using a 32-channel head coil with 17 slices acquired at a 2.75 × 2.75 × 7 mm^3^ resolution (multi-slice single-shot two-dimensional echo-planar imaging). The field-of-view was 220 × 220 mm^2^ and a sensitivity encoding (SENSE) factor of 2.5 was used. TR/TE = 4108/15 ms with two inversion pulses at 50 and 1150 ms for background suppression, applied during the post labeling delay of 1600 ms. Spectral presaturation inversion recovery (SPIR) was performed to suppress the lipid signal. The sequence cycled through four different labeling combinations (*A*_C_ *V*_C_, *A*_L_ *V*_L_, *A*_C_ *V*_L_ and *A*_L_ *V*_C_) and nine averages were acquired for all ten gradient amplitudes of *V*_enc_ and *A*_enc_. This resulted in a total scan duration of 52 min for both scans together (4 × 10 × 9 × 2 = 720 acquisitions).

### Image post-processing

The unsubtracted ASL-scans were realigned and the time series were corrected for motion with Motion Correction FMRIB’s Linear Image Registration Tool (MCFLIRT) [13, 14] with a six-parameter rigid transformation in Oxford Centre for Functional MRI of the Brain (FMRIB)’s Software Library (FSL) [15]. The anatomic *T*_1_-weighted scan of each subject was segmented into three different tissue types (grey matter (GM), white matter (WM), and cerebral spinal fluid (CSF) probability maps) using the FMRIB’s automated segmentation tool (FAST, FSL, Oxford, UK). The ASL-images are subtracted according to the combinations as described in Fig. 1. The *T*_1_-weighted image was registered to the average ASL-map of AccASL, VS-ASL and AccASL & VS-ASL. A binary GM mask was generated using a threshold of 75% GM probability in Matlab. The subtractions of the different labeling combinations were normalised by the average signal intensity in the GM for the SNS-ASL contrast that was kept constant (i.e. either VS-ASL or AccASL): so when the *V*_enc_ was varied, the signal was divided by the average AccASL signal in the GM. The amount of overlap in labeling by the combined sequential labeling with the Acc- and VS-module (*A*_C_ *V*_C_ − *A*_L_ *V*_L_) was determined by the sum of the separate labeling modules minus the sequential labeling, divided by the labeling module with the constant cut-off, expressed with the following formula when *V*_enc_ is constant:

1 $${\text{Overlap }} = \left( {\left( {\left( {A_{\text{C}} V_{\text{C}} {-}A_{\text{L}} V_{\text{C}} } \right) + \left( {A_{\text{C}} V_{\text{C}} {-}A_{\text{C}} V_{\text{L}} } \right)} \right) - \left( {A_{\text{C}} V_{\text{C}} - A_{\text{L}} V_{\text{L}} } \right)} \right)/\left( {A_{\text{C}} V_{\text{C}} {-}A_{\text{C}} V_{\text{L}} } \right).$$

## Results

**Fig. 2:**
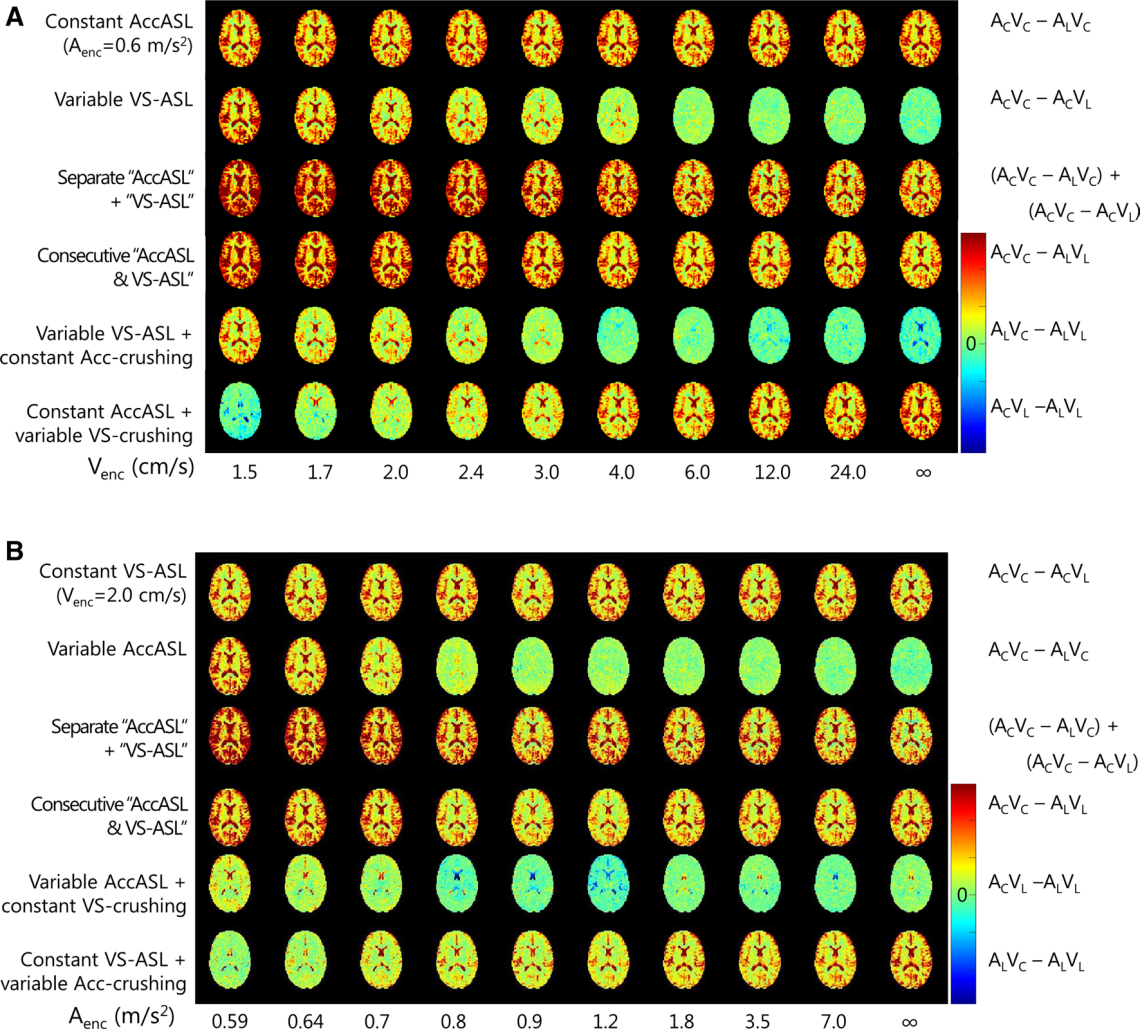
A representative set of single slice ASL maps (in arbitrary units) acquired at different cut-off velocities (**a**) and accelerations (**b**). The* top row* was acquired with a constant cut-off acceleration of 0.6 m/s^2^ (**a**) and cut-off velocity of 2 cm/s (**b**) and was depicted with a *similar number* of averages to illustrate the reproducibility of the measurements

In Fig. 2 a representative set of single slice ASL maps is shown for the different *V*_enc_s and *A*_enc_s (as presented in Table 1). The top row of Fig. 2a shows independent measurements of AccASL, i.e. (*A*_C_ *V*_C_ − *A*_L_ *V*_C_) with constant cut-off acceleration of 0.59 m/s^2^ and the same number of averages as the images in the other rows, to serve as a reference of reproducibility and to enable easy comparison. The velocity selective module is always executed in control condition (*V*_C_), so the varying scale of *V*_enc_s of the label condition as depicted on the horizontal axis has no influence on these measurements. Similarly, Fig. 2b shows in the top row VS-ASL images with a constant cut-off velocity of 2 cm/s (*A*_C_ *V*_C_ − *A*_C_ *V*_L_). For the maps with a variable cut-off velocity (second row of Fig. 2a) or acceleration (second row of Fig. 2b) the lower the cut-off velocity (acceleration), the more signal was labeled, whereas for a cut-off velocity (acceleration) of infinity only noise was measured.

Both for the variable cut-off velocity and acceleration the sum of the separate AccASL and VS-ASL ((*A*_C_ *V*_C_ − *A*_L_ *V*_C_) + (*A*_C_ *V*_C_ − *A*_C_ *V*_L_), third row of Fig. 2) has a slightly higher signal intensity than the jointly acquired AccASL and VS-ASL (*A*_C_ *V*_C_ − *A*_L_ *V*_L_, fourth row of Fig. 2). The ASL-maps with constant acceleration crushing (fifth row of Fig. 2a; similar to images with constant velocity crushing in Fig. 2b), the signal increases for decreasing cut-off, but the signal is less than without Acc-crushing (second row). In the bottom row of Fig. 2a (similar to images with variable acceleration crushing in the bottom row of Fig. 2b), AccASL is shown with variable velocity crushing and therefore, the rightmost image is similar to the images of the top row.

**Fig. 3:**
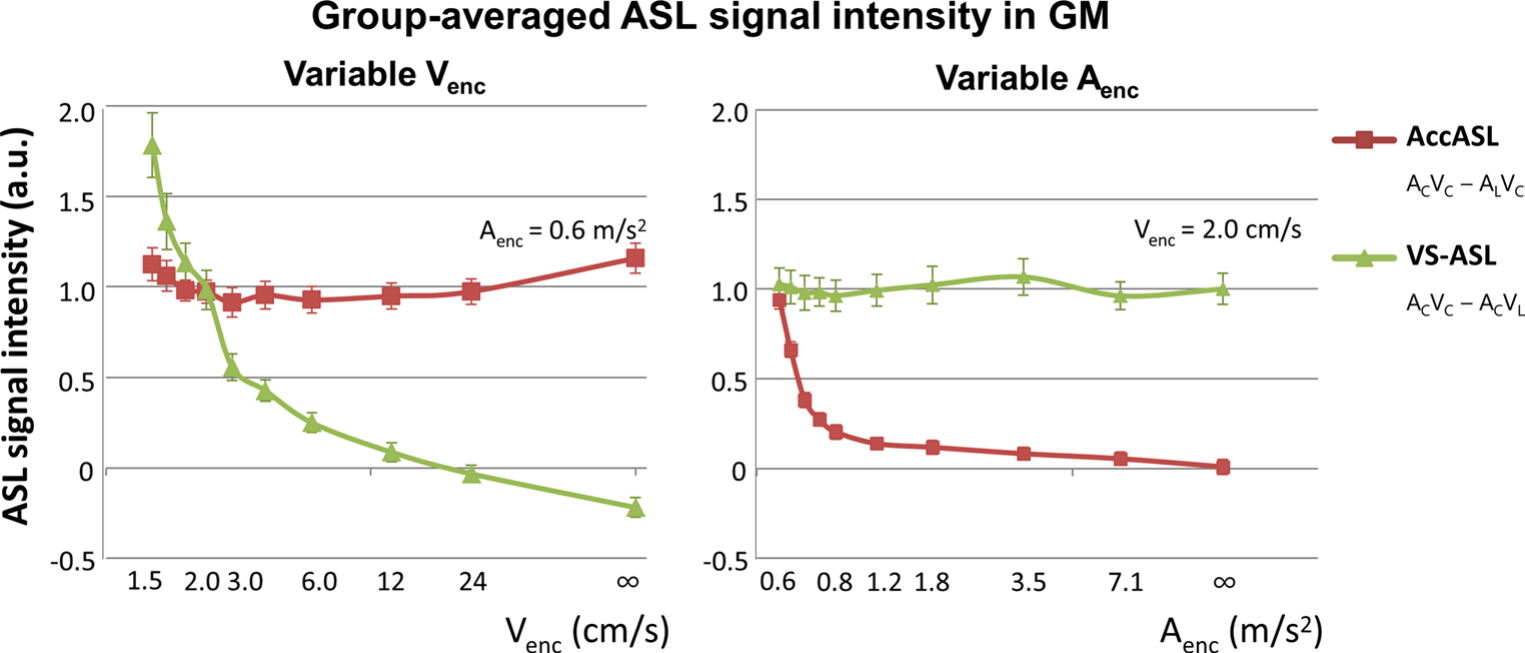
Group-averaged ASL signal intensities in GM of AccASL (*A*_C_ *V*_C_ − *A*_L_ *V*_C_,* red*) and VS-ASL (*A*_C_ *V*_C_ − *A*_C_ *V*_L_,* green*). For the variable *V*_enc_ (*left*) the signal was normalised to the average AccASL GM signal and for the variable *A*_enc_ (*right*) normalised to the average VS-ASL GM signal. In VS-ASL with a single labeling module a venous label is also included, while for AccASL the signal is mainly arterial. *Error bars* indicate the standard error of the means as calculated over all subjects. Note that the constant signal was also depicted at all cut-off velocities/acceleration with a *similar number* of averages to illustrate the reproducibility of the measurements

The group-average signal in grey matter for variable *V*_enc_ and *A*_enc_ are shown in Fig. 3. The signal is normalised by dividing through the constant signal, i.e. the mean GM signal of the top row of Fig. 2a (or b). Note that this constant signal serves as a reference experiment and was depicted at all cut-off velocities/accelerations with a similar number of averages for comparison, thereby also showing the reproducibility of the measurements. For the VS-ASL (*A*_C_ *V*_C_ − *A*_C_ *V*_L_) in the left graph of Fig. 3 at the highest *V*_enc_ no label is created and when the *V*_enc_ is decreased, more signal is created, up to 178% of the AccASL signal strength (*A*_enc_ = 0.59 m/s^2^) in GM for a *V*_enc_ of 1.5 cm/s. For the AccASL (*A*_C_ *V*_C_ − *A*_L_ *V*_C_) in the right graph of Fig. 3 at the highest *A*_enc_ no label is created and the more the *A*_enc_ is lowered, the more signal is created, up to 94% of the VS-ASL signal strength (*V*_enc_ = 2 cm/s) in GM for a *A*_enc_ of 0.59 m/s^2^.

**Fig. 4:**
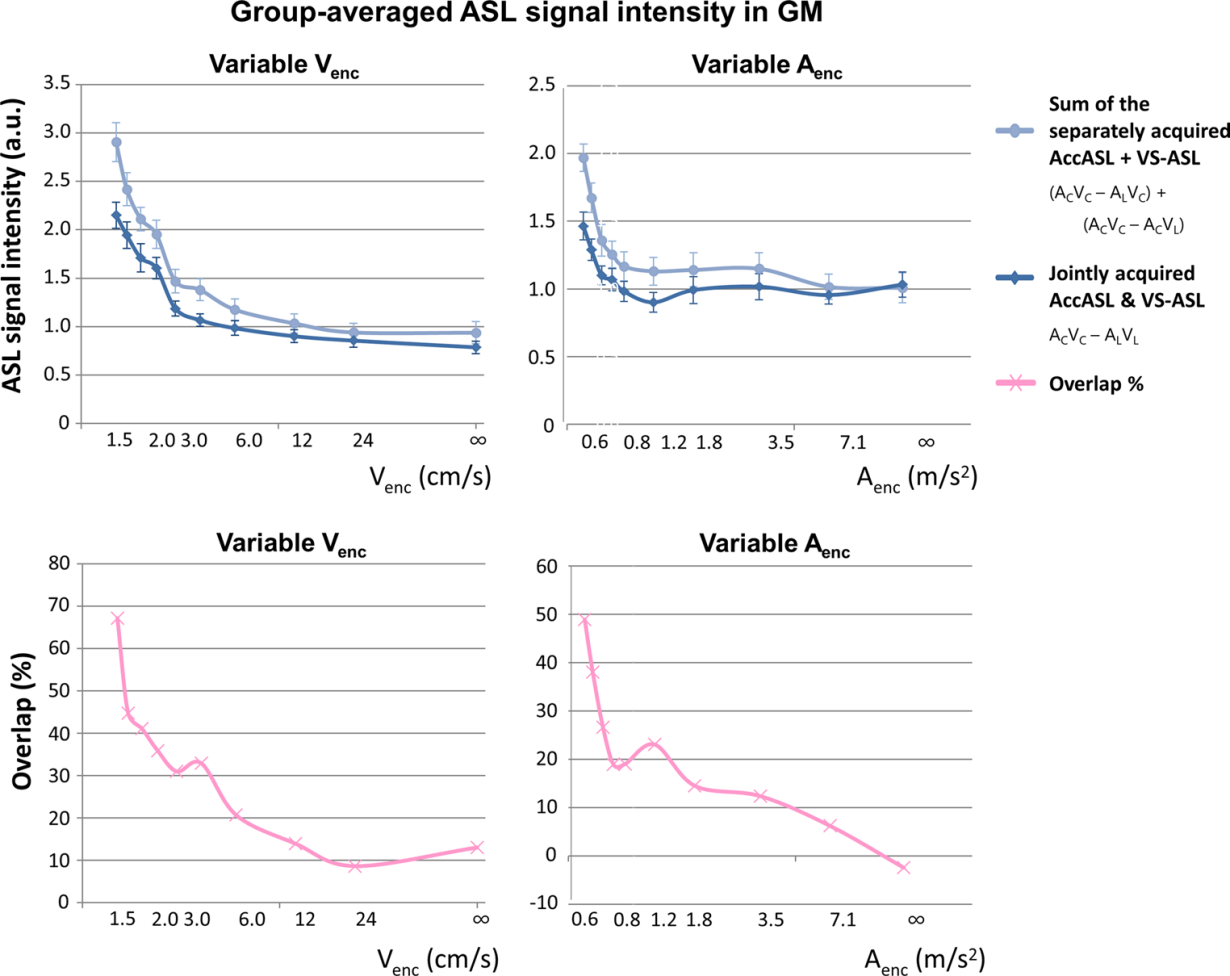
Group-averaged ASL signal intensities in GM (*top images*) of the sum of separate AccASL and VS-ASL (*A*_C_ *V*_C_ − *A*_L_ *V*_C_ + *A*_C_ *V*_C_ − *A*_C_ *V*_L_, *light blue*) and the joint subsequent acquisition of AccASL and VS-ASL (*A*_C_ *V*_C_ − *A*_L_ *V*_L_, *dark blue*). For the variable *V*_enc_ (*left*) the signal was normalised to the average AccASL GM signal (i.e. the mean GM signal of *A*_C_ *V*_C_ − *A*_L_ *V*_C_, *red* in *left image* of Fig. 3) and for the variable *A*_enc_ (*right*) normalised to the average VS-ASL (*A*_C_ *V*_C_ − *A*_C_ *V*_L_, *green* in *right image* of Fig. 3) GM signal. The *pink line* (*bottom images*) is the percentage overlap of the signal created with the variable labeling module to the signal of the constant labeling module. The *error bars* indicate the standard error of the mean as calculated over all subjects

The difference between the combined sequential labeling with the Acc- and VS-modules (*A*_C_ *V*_C_ − *A*_L_ *V*_L_) and the sum of the signal of both labeling modules acquired separately (VS-ASL + AccASL, i.e. (*A*_C_ *V*_C_ − *A*_L_ *V*_C_) + (*A*_C_ *V*_C_ − *A*_C_ *V*_L_)) is a measure for the amount of spins that are saturated by both labeling modules. In Fig. 4 it is shown that at the highest cut-off there is hardly any difference between the two labeling combinations, which can be explained by the fact that there is hardly any labeling achieved by either the velocity (left) or the acceleration selective module (right). However, independent of which component of the labeling is varied, the sum of separately acquired signals is always higher than the combined labeling (up to 35% higher signal intensity at the lowest cut-off value).

**Fig. 5:**
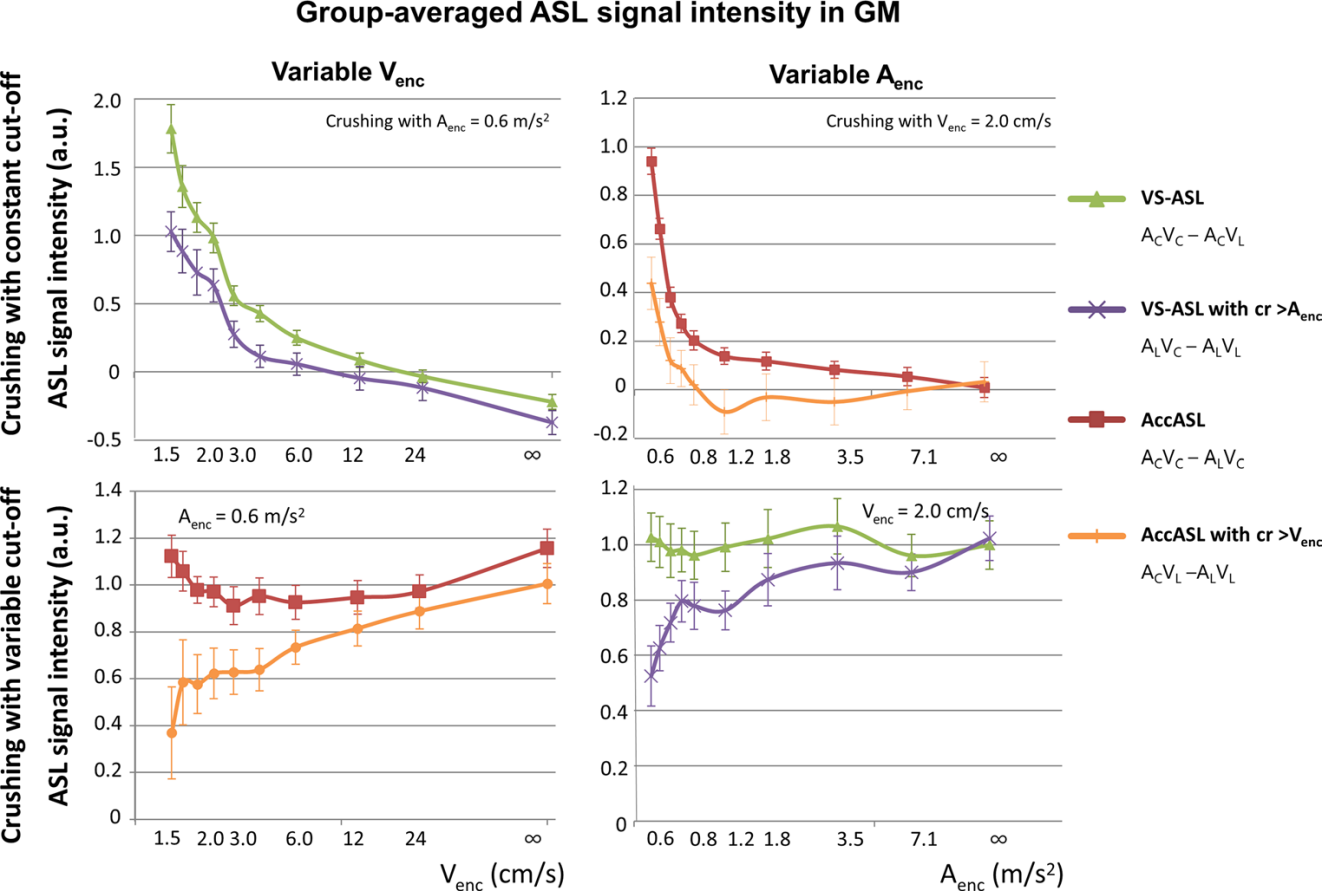
Group-averaged ASL signal intensities in GM of “AccASL” (*A*_C_ *V*_C_ − *A*_L_ *V*_C_, *red*), “VS-ASL”(*A*_C_ *V*_C_ − *A*_C_ *V*_L_, *green*), “VS-ASL with crushing due to AccASL” (*A*_L_ *V*_C_ − *A*_L_ *V*_L_, *purple*) and “AccASL with crushing due to VS-ASL” (*A*_C_ *V*_L_ − *A*_L_ *V*_L_, *orange*). For the variable *V*_enc_ (*left*) the signal was normalised to the average AccASL GM signal and for the variable *A*_enc_ (*right*) normalised to the average VS-ASL GM signal by dividing through the constant signal. In the* top row* the crushing was performed with a constant cut-off and the labeling was performed with different gradient strengths. In the* bottom row* the crushing was performed with a variable cut-off, while the labeling was kept constant. The *error bars* indicate the standard error of the means of all subjects

To discriminate the amount of label that was uniquely labeled by one of the labeling modules from a label jointly created by both labeling modules, the effect of crushing with one module on the labeling by the other module was studied. In the top graphs of Fig. 5 the signal intensity in GM is shown when labeling had a variable cut-off and additional crushing was constant, where the difference between both labeling methods indicates the overlap in labeling. In the top left graph of Fig. 5 the effect of crushing with a constant *A*_enc_ of 0.59 m/s^2^ on the variable *V*_enc_ is clearly visible: a decrease up to 42% in the signal intensity at a *V*_enc_ of 1.5 cm/s was observed due to the crushing. A similar effect can be seen in the top right graph of Fig. 5, showing the effect of crushing with a constant *V*_enc_ of 2 cm/s on the variable *A*_enc_.

In the bottom graphs the signal intensity in GM is displayed with a constant cut-off for labeling and a variable cut-off for crushing. The difference between both curves indicates the overlap in labeling. A decrease of up to 36.9% of the AccASL signal is possible, with the highest *V*_enc_ crushing, as shown in the bottom left graph of Fig. 5. For the labeling with a constant cut-off velocity with crushing with a variable cut-off acceleration (bottom right graph of Fig. 5) a plateau with a signal intensity of 0.78 was reached between *A*_enc_ = 0.79 and 1.2 m/s^2^, after which a steep decline in signal intensity was measured due to crushing with lower cut-off accelerations. By crushing with a variable *A*_enc_ the signal intensity was decreased up to 52.5%.

## Discussion

For the recently introduced AccASL it is unknown where in the vasculature the label is created. Several possible sources of the label origin have been mentioned previously, including cardiac cycle fluctuations, general flow acceleration/deceleration in the vasculature and tortuosity of the vessels leading to rapid changes in blood flow directions. Therefore, it has been suggested that the label could originate at both macro- and microvascular levels and that the signal created with AccASL includes both CBF and CBV-weighting [4]. All of these effects, besides tortuosity, could explain the observation that with AccASL much more arterial than venous blood is labeled. The aim of this study was to obtain more insight into the origin of the labeling mechanism in AccASL by combining this method with a VS-module.

The most important findings of this study are that the label created with AccASL has a large overlap in vascular region with VS-ASL (approximately 50%), but also originates from smaller vessels closer to the capillaries. This shows that AccASL is able to label spins both in the macro-, meso-vasculature, as well as in the microvasculature.

The indication that AccASL shares, for a significant part, the same label origin as VS-ASL can be seen from Fig. 4, which shows that the sum of separately acquired AccASL and VS-ASL scans ((*A*_C_ *V*_C_ − *A*_L_ *V*_C_) + (*A*_C_ *V*_C_ − *A*_C_ *V*_L_), in light blue) results in a higher signal intensity compared with sequentially acquired AccASL and VS-ASL (*A*_C_ *V*_C_ − *A*_L_ *V*_L_, in dark blue). This difference reaches up to 35% for the lowest cut-off. This argument is based on the fact that spins can be saturated only once, i.e. when AccASL and VS-ASL would label exactly the same spins in the vascular tree, the application of the VS-module directly after the AccASL would have no effect and equal signal intensity would be measured. When assuming the signal intensity of AccASL, i.e. (*A*_C_ *V*_C_ − *A*_L_ *V*_C_), to be one, the intensity of VS-ASL (*A*_C_ *V*_C_ − *A*_C_ *V*_L_) as well as the signal of sequentially acquired AccASL and VS-ASL (*A*_C_ *V*_C_ − *A*_L_ *V*_L_) would also be one, whereas the sum of separately acquired AccASL and VS-ASL scans ((*A*_C_ *V*_C_ − *A*_L_ *V*_C_) + (*A*_C_ *V*_C_ − *A*_C_ *V*_L_)) would yield twice the amount of signal. When looking at Fig. 4 at the point where AccASL and VS-ASL each create a similar amount of label (i.e. the point where the sum of separately acquired VS-ASL and AccASL equals 2.0, i.e. at a *V*_enc_ of 2.4 cm/s in the left graph and at an *A*_enc_ of 0.59 m/s^2^ in the right graph), consecutive labeling (*A*_C_ *V*_C_ − *A*_L_ *V*_L_) shows only 1.6 and 1.5 times the reference value, implying an overlap in signal of, respectively, 40 and 50%. However, the fact that the overlap was much less than 100% indicates that the two SNS-ASL modules also label spins in different parts of the vasculature. This is further confirmed by the significantly higher signal (up to 120% higher signal for the lowest *V*_enc_) when performing a VS-module immediately after AccASL (dark blue line from the left graph of Fig. 4) as compared with just AccASL (red line from the left graph of Fig. 3). The comparison of performing the VS-module immediately after AccASL (dark blue line from the right graph of Fig. 4) compared with only VS-ASL (green line from the right graph of Fig. 3) also showed a significantly higher signal, up to 50% for the lowest *A*_enc_. The higher signal points to the fraction of spins that could additionally be labeled by the other labeling module. When interpreting these findings, one has to keep in mind that in this study only a single velocity selective labeling module was performed, instead of using a second labeling module just before or during the imaging read out, as is part of the original VS-ASL methodology [3]. An isolated velocity selective labeling block will also generate a venous signal, and in previously research we showed that the amount of venous label generated by a single velocity selective module is much more than that of an acceleration module [4]. In the original VS-ASL sequence, this venous signal will be subsequently excluded by the second VS-module [3, 4]. Some of the difference in signal intensity between the velocity and acceleration modules, therefore, will be from venous spins that will be more affected by the velocity encoding than the acceleration encoding. This can explain why adding a VS-module to the AccASL scan resulted in much more signal increase than when adding an Acc-module to the VS-ASL scan.

Further confirmation that a significant component of signals created by the Acc- and VS-module originates from different parts of the vasculature can be deduced from the results in which labeling was combined with crushing. When crushing with a constant *V*_enc_ of 2 cm/s the signal intensity in the GM decreased, as shown in the top right graph from Fig. 5. For *A*_enc_s larger than 0.79 m/s^2^ the crushing is effective, as shown by the fact that the orange curve is almost horizontal. This implies that for these relatively weak acceleration strengths, only spins are labeled that flow faster than 2 cm/s (the *V*_enc_ of the crushing). However, when the *A*_enc_ is decreased below 0.79 m/s^2^, crushing is no longer as effective as a steep increase in signal is found, proving that the additional label created by the stronger gradients in the acceleration module originates from spins with a velocity smaller than 2 cm/s, i.e. closer to the capillaries. For constant AccASL with variable crushing by a VS-module (lower left graph in Fig. 5), it can be seen that for a *V*_enc_ smaller than 1.5–3 cm/s the slope of the signal intensity curve gets steeper. Velocities lower than 3 cm/s are found in the arterioles, where the flow velocity rapidly decreases towards the capillaries, again pointing to significantly labeling of spins in the capillaries by AccASL. Furthermore, it should be noted that by crushing performed by the acceleration selective labeling module, as in the lower right graph of Fig. 5, a label created by the velocity selective module that also resides in the venous compartment will not be affected, which could be an explanation for the smaller effect of the crushing. The other way around, with crushing by the velocity selective module the venous signal will be saturated twice, although this will have hardly any influence on the AccASL signal, since this label will mainly be in the arterial compartment.

For AccASL a lower *A*_enc_ could have been chosen, to label even further into the vascular tree. This was, however, not possible due to hardware restrictions of the scanner limiting the gradient amplitudes (*G*) to these values. Smaller *A*_enc_s could be achieved by increasing the time between the gradients (*Δ* and *τ*) or increasing the gradient duration (*δ*), however both options would lead to an increased diffusion sensitivity. In this study, we opted for employing the same *δ* and *Δ* for all SNS-modules and varying only the gradient polarity (velocity versus acceleration) and gradient strength. Increasing the gradient strength will lead to more diffusion weighting with a maximum b-value of 2.4 s/mm^2^ for AccASL with *G* = 30 mT/m, *Δ* = 17.5 ms and *δ* = 1 ms and 1.4 s/mm^2^ for VS-ASL with *G* = 20 mT/m, *Δ* = 17.5 ms and *δ* = 1 ms. For typical values of brain apparent diffusion coefficient, 0.8 × 10^−3^ mm^2^/s in GM and 2.5 × 10^−3^ mm^2^/s in CSF, the *b*-values in this study could theoretically cause a systematic difference between labeled and control images of less than 1% [3, 4]. Furthermore, the influence of contamination from eddy current and/or diffusion effects was evaluated on a liquid gel phantom (shown in the supplementary material). This showed that the intensity of the artefacts was approximately only 0.1% of the *M*_0_-signal (acquired with the same imaging parameters as for the ASL scans, but no labeling performed) in the same voxels for the constant labeling module. An increase in signal was found when increasing the gradient strengths, up to 0.23% of the *M*_0_-signal for the lowest *V*_enc_ and *A*_enc_. This indicates that contamination from eddy current/diffusion effects is very limited. Also, as expected these artefacts show as large regions of signal change, which would affect the white matter/gray matter contrast significantly in the perfusion maps of the volunteers if it were a significant effect. No such effects were observed in the in vivo data in Fig. 2.

This study has some limitations. First of all, no cardiac triggering was applied during the measurements. The pulsatile effects from the cardiac cycle will be averaged over the dynamics and will have no specific influence on the data. Secondly, it is important to realize that the presented results are not independent from each other, since they are all calculated by adding and subtracting four types of images. For example, the image with both labeling modules in control condition (*A*_C_ *V*_C_), is used to calculate AccASL, VS-ASL, the joint AccASL & VS-ASL signals, whereas in the calculation of the sum of the separate AccASL and VS-ASL maps it is even included twice. This implies that noise and artefacts in this map will be present in the results of all these maps, which might result in erroneous conclusions. Similarly, the other three acquisitions (*A*_L_ *V*_L_, *A*_C_ *V*_L_, *A*_L_ *V*_C_) are used in multiple calculations. Thirdly, in the control condition a small gradient amplitude was used similar to that proposed for VS-ASL with linear RF pulses by Duhamel et al. [12] to reduce the errors resulting from imperfect flip angles. In this study adiabatic RF-pulses were employed, and in the labeling condition with varying gradient strengths a zero amplitude starting value was chosen. Therefore, no gradients in the control condition would have been a more optimal choice, although the amplitude (5% of the constant cut-off) of these gradients was low enough to not significantly influence the results. Finally, the gradients of the labeling modules were applied in only a single, similar direction, whereas blood flowing through the branches of the vasculature will be in all directions and will also change direction; especially in the capillaries the flow will be in random directions. So, the assumption that all spins with a flow velocity above the *V*_enc_ are labeled with VS-ASL is not true, since only spins flowing in the *z*-direction with a velocity above the *V*_enc_ will be labeled. Nevertheless, the same directional dependency is present in the VS-ASL as well as in the AccASL labeling module, making the results of this study still valid.

## Conclusion

In conclusion, AccASL is able to label spins in both the macro- as well as the micro-vascular region and the label created with AccASL has a large overlap in the vascular region with VS-ASL, but depending on the *A*_enc_ the label from AccASL could also originate from regions closer to the tissue. This might imply that AccASL could be preferable over VS-ASL in patients with pathologies showing prolonged arrival times, due to the label being closer to the tissue.

## Electronic supplementary material

Below is the link to the electronic supplementary material.

Supplementary material 1 (DOCX 1386 kb)
