# Supplementary material for: Insight into the labeling mechanism of acceleration selective arterial spin labeling
Source: MAGMA. 2016 Oct 27;30(2):165–74. doi: 10.1007/s10334-016-0596-6 (PMC5364255; doi:10.1007/s10334-016-0596-6)
Supplement: Supplementary file 1 — Supplementary material 1 (DOCX 1386 kb) [file 10334_2016_596_MOESM1_ESM.docx]

**Supplementary material**


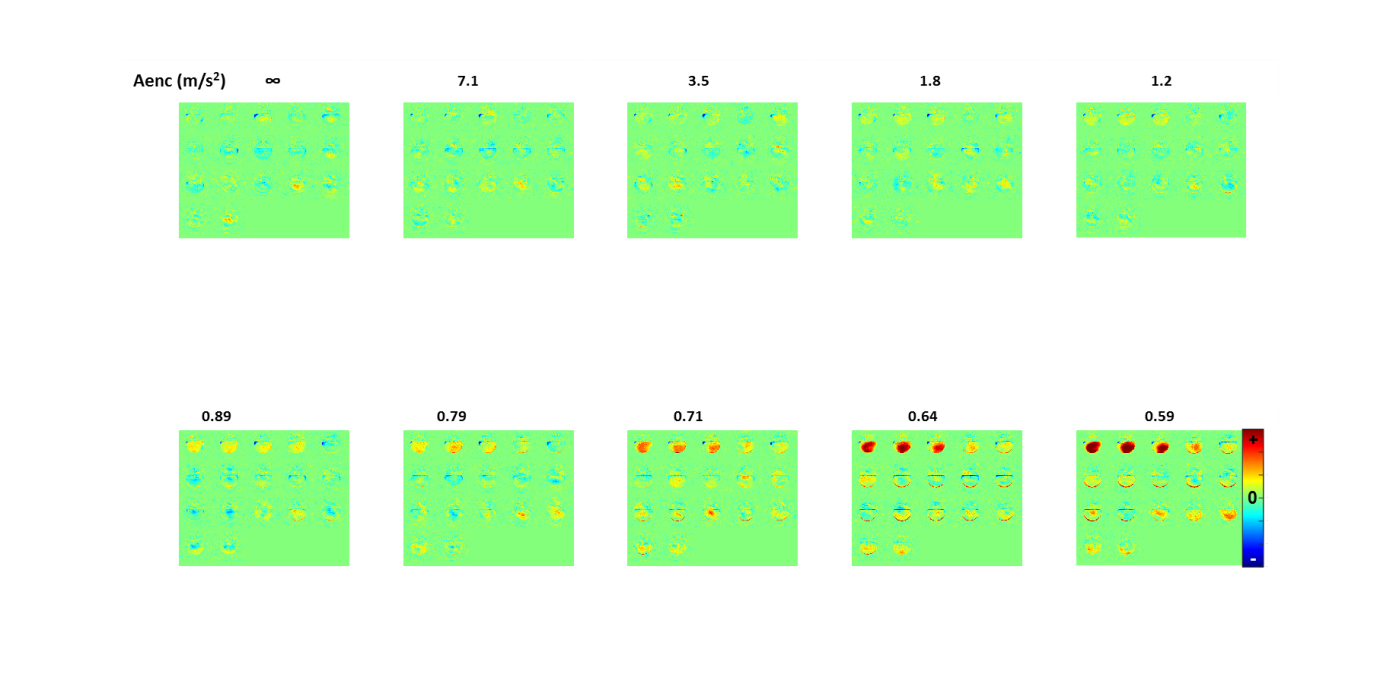

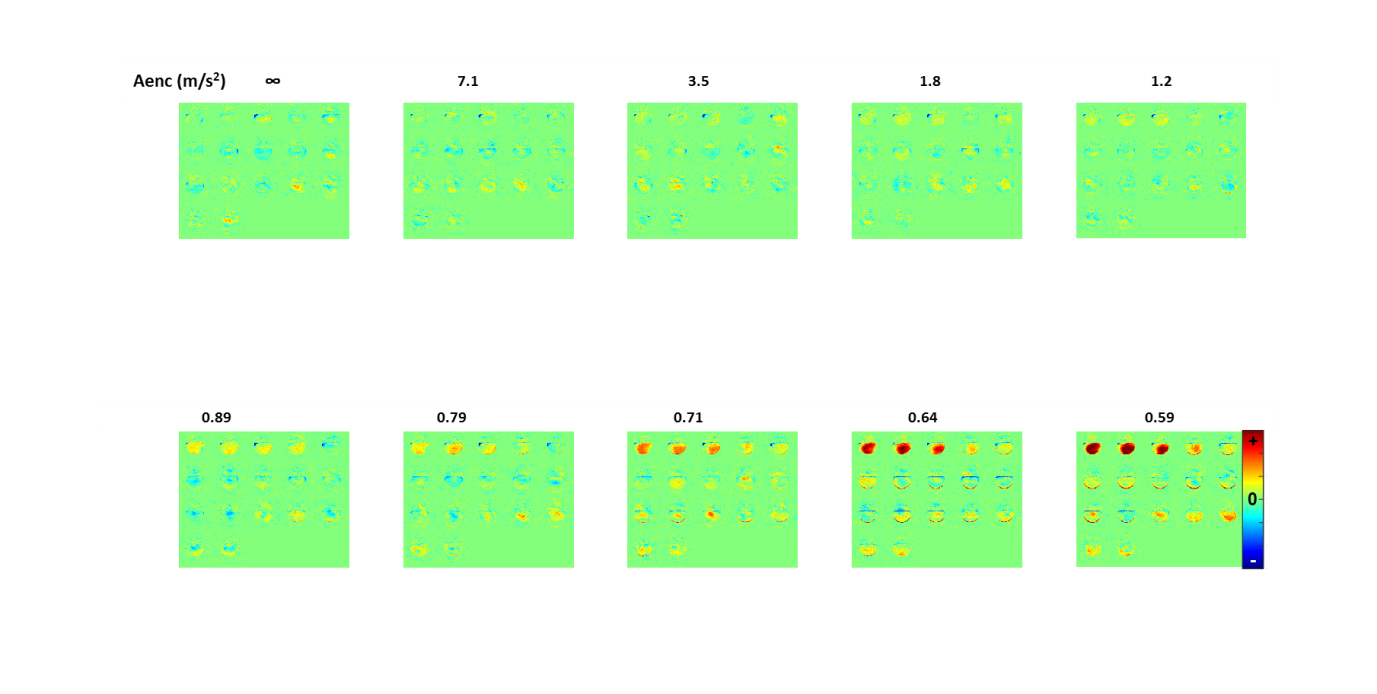


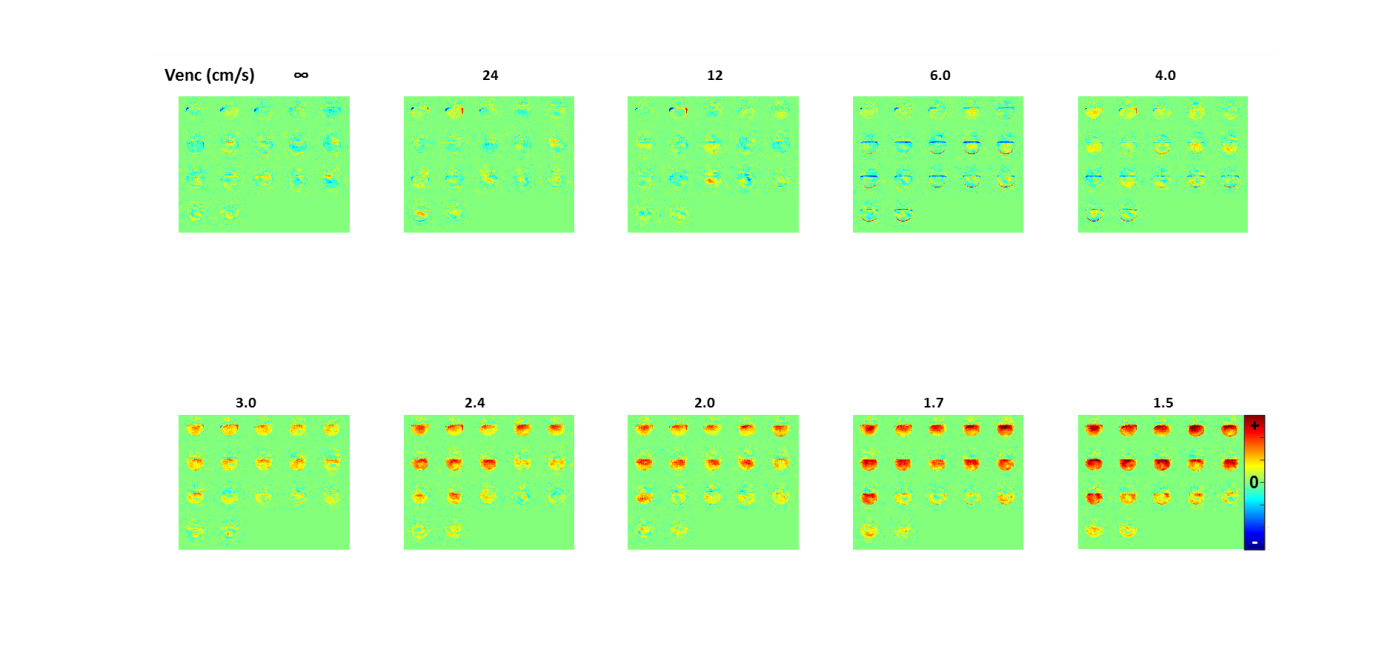

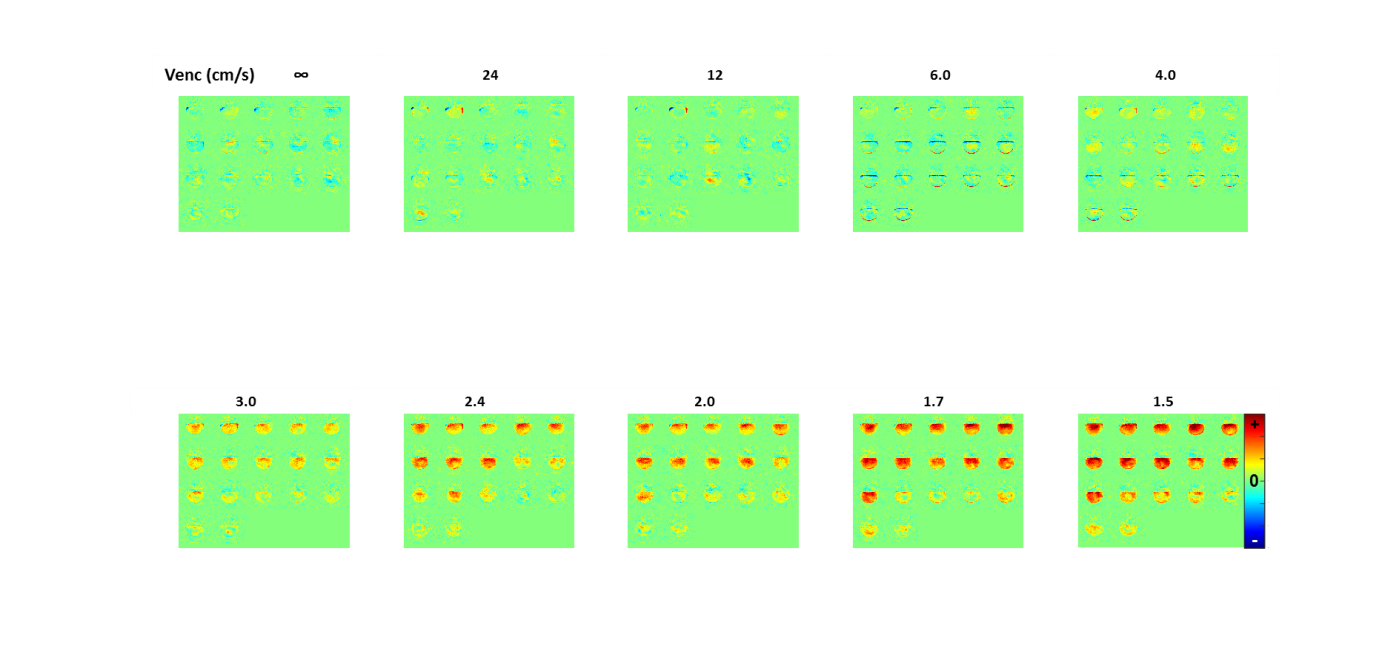


**Figure S1.** AccASL (top) and VSASL (bottom) maps of a gel phantom measured with variable A_enc_ and V_enc_ respectively and keeping the other labeling module in control condition. Measurements were performed with same settings as the *in vivo* measurements.


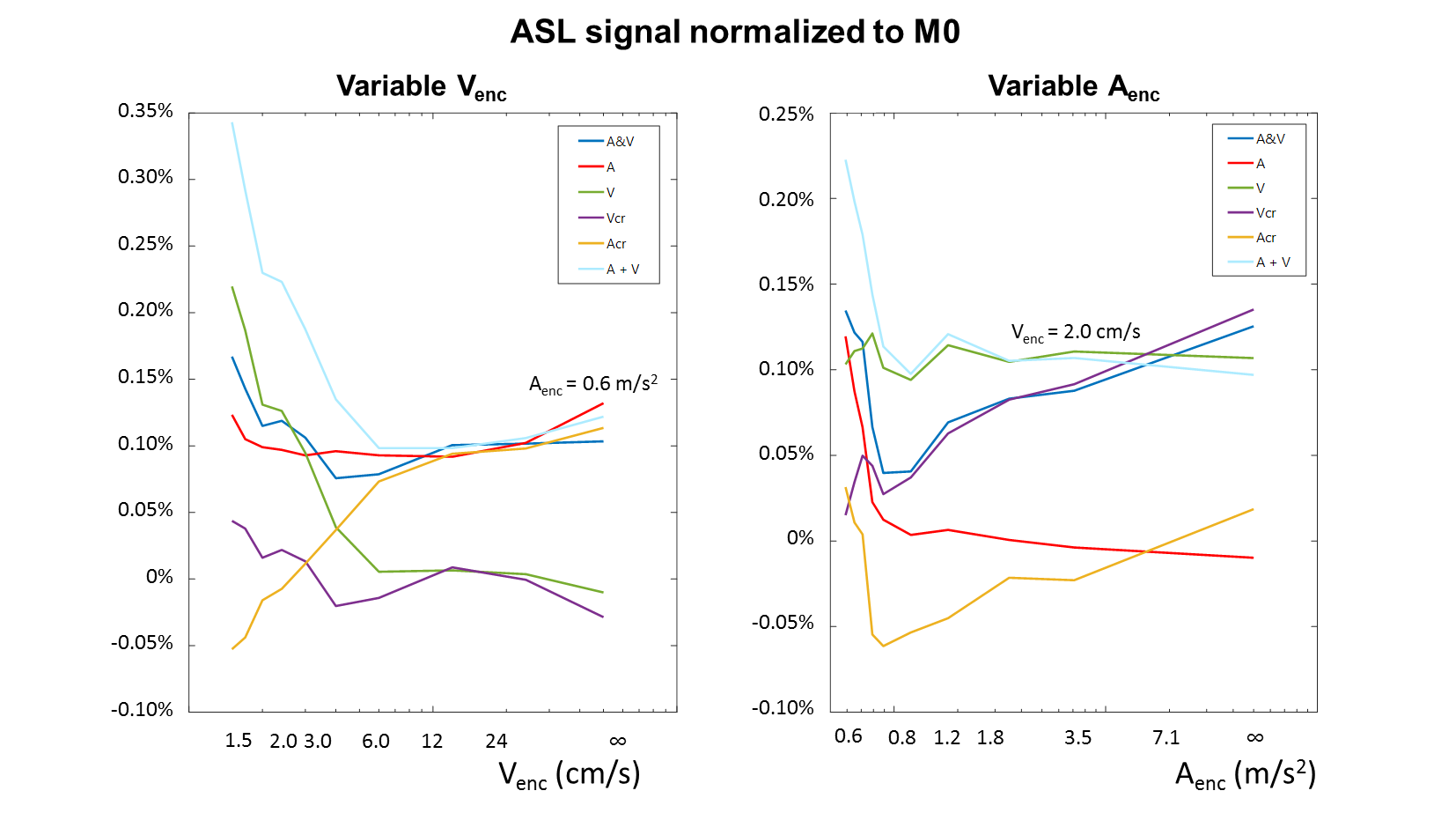


**Figure S2.** Average signal intensity within the gel phantom measurements of figure S1 with variable VSASL labeling module (left) and variable AccASL labeling module (right), while keeping the other labeling module in control condition. Signal was normalized by dividing through the M­_0_-signal, acquired using the same imaging parameters as for the ASL scans without labeling. For the constant labeling module the intensity of the artefacts was approximately only 0.1% of the M_0_-signal. An increase in signal was found when increasing the gradient strengths, up to 0.23% of the M_0_-signal for the lowest V_enc_ and A_enc_.
